# Climate Change and Adolescent Girls’ Sports: A Scoping Review and Framework-Based Exploration of Emerging Barriers and Recommendations

**DOI:** 10.3390/ijerph22121764

**Published:** 2025-11-21

**Authors:** Jayda Hylton-Pelaia, Satveer Dhillon, Caroline Barakat

**Affiliations:** 1 Faculty of Health Sciences, Ontario Tech University, 2000 Simcoe Street North, Oshawa, ON L1G 0C5, Canada; jayda.hyltonpelaia@ontariotechu.net (J.H.-P.); caroline.barakat@ontariotechu.ca (C.B.); 2 Department of Geography and Environmental Management, University of Waterloo, Waterloo, ON N2L 3G1, Canada

**Keywords:** climate change, sports, adolescent girls, ecologism, Individual × Environment (I×E) framework

## Abstract

Climate change poses growing challenges to youth sports participation, but adolescent girls face disproportionate and compounding vulnerabilities. These arise from sex-specific physiological factors, sociocultural constraints, and institutional inequities that uniquely and disproportionately impact girls. These challenges are especially concerning considering the numerous health and well-being benefits of sports participation. However, there is a notable lack of research examining the specific impacts of climate change on adolescent girls’ sports participation, as well as evidence-informed strategies to mitigate these effects. The aim of this study is to (1) conduct a scoping review to better understand the impacts of climate change on adolescent girls’ sports participation and (2) examine the relationship between climate change and adolescent girls’ involvement in sports by integrating Ecologism and the Individual × Environment (I×E) frameworks. A search was conducted using four databases (PubMed, Scopus, SPORTDiscus and Web of Science), and a gray literature search was performed on Google. The search was limited to studies focusing on how climate change or weather variables impacted adolescent girls’ physical activity levels or sports participation. Studies must have been written in English, and all geographical regions were included. In total, 26 studies met the inclusion criteria. These findings were then analyzed by integrating Ecologism, which promotes sustainable infrastructure, and the Individual × Environment (I×E) framework, which highlights interventions tailored to individual and environmental interaction. Recommended strategies include climate-resilient facility design, equity-focused funding models, participatory research, and coordinated efforts from public health units and urban planning stakeholders. By integrating these frameworks, the paper proposes a comprehensive set of interventions that address both systemic ecological challenges and individual-level barriers, aiming to foster climate-resilient sports environments for adolescent girls.

## 1. Introduction

Climate change is one of the most pressing global challenges of today, with far-reaching consequences for ecosystems, human health, and societal well-being [1,2]. Rising temperatures, increased frequency of extreme weather events, and worsening air quality are just a few of the environmental changes reshaping how communities operate. Among its numerous consequences, climate change has significant implications for human health, particularly for vulnerable populations, including adolescents. For example, direct and indirect effects of climate change increase the risk of asthma, respiratory illnesses, diarrheal diseases, and vector-borne diseases [3]. Poor air quality and heatwaves are increasingly common, directly limiting adolescents’ ability to participate in safe physical activity [4].

In this context, sports participation, which is a critical component of youth development and well-being, is increasingly threatened by the environmental disruptions caused by climate change. Participating in sports during adolescence is associated with numerous benefits for physical, psychological, and social health, including improved physical fitness, enhanced mental well-being, improved metabolic health, and cognitive advantages [5,6].

Despite these benefits, research indicates that a troublingly high proportion of youth, approximately 70%, drop out of organized sports by the age of 13 [7,8]. This issue is more pronounced among girls, who participate in sports less frequently than boys at all age levels, with the gap widening during adolescence [8]. While prior research has examined sociocultural, structural, and interpersonal barriers [9,10], there is limited exploration of how emerging environmental factors, particularly climate change, compound these barriers for sports participation. Risks such as extreme heat, air pollution, and unsafe playing conditions are increasingly relevant and may uniquely affect girls’ ability to participate in sports [11].

### 1.1. Climate Change as an Environmental Determinant of Health

#### 1.1.1. Impacts of Climate Change on Sport Environments

Climate change is impacting the physical and social environments where sports and physical activities occur, introducing risks that disrupt participation and health. One of the most prevalent effects is the increase in extreme weather events. Heatwaves, for example, not only jeopardize the safety of athletes by exposing them to heat-related illnesses such as heatstroke and dehydration, but also limit the usability of outdoor sports facilities due to unsafe conditions [12]. Prolonged periods of high temperatures create an environment where even a short duration of outdoor activity can result in overheating [13]. Flooding and storms further damage sports infrastructure, rendering fields, tracks, and courts unusable [12]. There are also several indirect impacts due to climate change, such as increased exposure to allergens and a higher risk of respiratory problems due to changing air quality [12,14]. These risks are higher for those participating in outdoor physical activity [12].

Beyond these impacts, the recent literature highlights other sport-specific consequences that extend beyond immediate health concerns. Sport seasons may be shortened or disrupted due to extreme weather events, which can interfere with athlete development [15]. To adapt, sport organizers may shift events to cooler months or different times of day, creating logistical challenges and inequities in facility access. Warming temperatures are also leading to the loss of natural sport environments, such as ski slopes, skating ponds, and open-water venues, fundamentally altering the types of sports available in certain regions [16]. Also, the quality and safety of existing sport surfaces are at risk, with turf warping, track melting, and ice instability increasing both injury risk and performance limitations [15].

Indoor sports are not immune to these pressures. Rising temperatures and extreme weather events drive increased energy demands for cooling systems, raising operational costs that can limit affordable access to facilities. On a global scale, climate change is prompting the relocation of major sporting events to cooler or less climate-affected regions, which can reduce opportunities for local communities’ exposure to elite-level competition and visible role models [15]. Ecosystem changes, such as the spread of invasive species or harmful algal blooms, are also impacting water-based sports, reducing both their safety and availability [15].

These broader sport environment consequences illustrate that climate change is not only a public health concern but also a disruptor of the sporting ecosystem from grassroots to elite levels. For adolescent girls, these disruptions intersect with existing participation barriers, such as inequitable resource allocation, gendered safety concerns, and reduced role model visibility, amplifying inequities in access, safety, and opportunity.

#### 1.1.2. Vulnerabilities of Adolescents

Adolescents are particularly vulnerable to the health impacts of climate change, especially when engaging in physical activity and sports. Physically, they are more susceptible to heat-related illnesses, such as heat exhaustion, dehydration, and heatstroke, due to their smaller body size and less efficient thermoregulation [17]. Rising temperatures cause increased stress on cardiovascular, respiratory, and metabolic systems—effects that can be especially dangerous in this age group [18]. For those with preexisting conditions, such as asthma or allergies, exposure to increasingly poor air quality—driven by higher levels of pollutants and allergens in the atmosphere—further elevates health risks during exercise [19].

Beyond physical health, the psychological impacts of climate change-related disruptions can affect adolescents. Loss of access to safe recreational spaces due to extreme weather or infrastructure damage can lead to decreased physical activity, feelings of isolation, and increased stress or anxiety [12]. For adolescent girls, these stressors are compounded by other barriers, such as the perception of sports as a lower priority for girls compared to boys during resource allocation [20]. In addition, adolescents tend to have different risk perception than adults, making them more prone to underestimate the dangers of being active in extreme heat [3,21]. This can increase the likelihood of overexertion or delayed cooling-down responses. This tendency is compounded by the authority dynamics in coach–athlete relationships, which may deter young athletes from voicing discomfort or requesting breaks—especially when experiencing early signs of heat stress [14,22]. Adolescent girls may be even less likely than boys to speak up in such situations, influenced by gender norms around deference and fear of being perceived as weak, thereby increasing their vulnerability [9,23].

### 1.2. Girls’ Sports Participation: Barriers

#### 1.2.1. Environmental Barriers

Environmental challenges posed by climate change create significant barriers to adolescent girls’ participation in sports, particularly outdoor activities (see Table 1). Extreme weather events—such as heatwaves, storms, and flooding—not only disrupt training schedules but also create unsafe conditions for athletes [12]. Rising temperatures intensify heat stress, which can be especially dangerous for younger participants who are less capable of thermoregulation [24]. Adolescent girls may be particularly affected due to physiological differences, including lower sweating efficiency and varying thermoregulary responses, which can make exercise in hot conditions more uncomfortable and discouraging [25,26].

Social and psychological factors intersect with these physiological challenges. Girls often report feeling uncomfortable with sweating, experiencing embarrassment around physical exertion, all of which can deter participation [23]. Increased exposure to UV radiation due to longer seasons and sunnier days also poses health risks. Girls may be more vulnerable because of uniform designs that expose more skin and beauty ideals that promote tanning, increasing cumulative sun damage over time [24,27,28]. For some, cultural or religious clothing requirements—such as wearing a hijab or long sleeved garments—may heighten heat stress by limiting evaporative cooling during exertion [3,29].

Extreme weather like storms and flooding damage physical infrastructure, such as sports fields and courts, leading to long-term degradation of community sports facilities and reducing access for young athletes [30]. Physiologically, the impact is universal, but adolescent girls are disproportionately affected because they are more likely to participate in underfunded community-based or school sport programs, which often lack the financial and infrastructural capacity for rapid recovery after extreme weather events [31].

Poor air quality, driven by rising levels of pollutants, further limits opportunities for physical activity. Active youth inhale more air—and with it, more pollutants—than their sedentary peers [32]. Adolescent girls may experience greater respiratory burden due to smaller airway diameters and higher rates of asthma incidence post-puberty [33]. These environmental risks can discourage parents from allowing their children, especially girls, to participate in sports, further amplifying the barriers to participation.

#### 1.2.2. Individual and Social Barriers

In addition to environmental obstacles, individual and social factors further limit adolescent girls’ ability to engage in sports (see Table 1). Adolescent girls report higher climate-related anxiety than boys, which can influence engagement and motivation in outdoor sports [12,20,34]. Preexisting conditions, such as asthma and allergies, are often exacerbated by climate change, making it more challenging for individuals with these conditions to actively participate in and thrive in sports [19]. Adolescent girls have been found to experience higher asthma prevalence post-puberty, potentially due to hormonal influences on airway reactivity [33]. Overall, research underscores that symptoms of health-related conditions such as asthma and eczema have been shown to disproportionately affect girls, particularly in poor weather conditions, high heat, or environments with high air pollution [35,36]. Similarly, with the increased UV radiation exposure, girls are at higher risk for photoaging, skin damage caused by the sun, and an elevated likelihood of developing skin cancers later in life [37].

The psychological impacts of climate-induced disruptions also present barriers. For example, interruptions to established routines caused by these disruptions can undermine motivation and reduce opportunities for social engagement and teamwork, which are vital benefits of sports participation [22]. To highlight, repeated cancelations of practices due to extreme weather conditions can lead to feelings of frustration and disengagement, as well as a diminished sense of connection with teammates and coaches, further discouraging consistent participation. Furthermore, to combat high temperatures, training sessions may need to be scheduled later in the evening, when the sun has set, potentially disrupting sleep patterns by causing girls to go to bed later [38]. Later rescheduling to avoid heat (e.g., evening practices) may also disproportionately affect girls, as safety concerns when traveling home at night can limit attendance [38]. Such scheduling can raise parental concerns about safety, potentially leading to restrictions on participation. Recent research has concluded that girls, but not boys, identify academic demands as a key reason for their lack of participation in sport programs [39]. Shifting activities to later hours can conflict with homework and study schedules, leaving less spare time for sport participation, and further limiting opportunities for engagement.

Social challenges, particularly gender biases, intensify the barriers girls face in sports participation amid climate change. Due to structural gender bias, girls’ programs often receive less funding and are deprioritized during climate adaptation efforts, with resources frequently directed toward facilities used by boys’ teams or professional leagues [40]. Furthermore, post-disaster recovery efforts tend to favor male-dominated sports infrastructure, sidelining female-centric initiatives [31]. Women and girls are underrepresented in sports governance, meaning adaptation strategies often fail to address female-specific needs in changing climates [31,40]. Scheduling inequities mean women’s professional teams often play midday during peak heat, raising the risk of climate-related cancelations, while men’s teams more often play in cooler evening hours [41]. Reduced visibility and representation of female athletes’ further limits role models for girls, eroding inspiration and reinforcing perceptions that girls’ sports are less valued [42]. In addition, adolescent girls are more likely to face the burden of caregiving during climate events, such as assisting family members during floods or droughts, which can lead to missed training sessions and long-term withdrawal from sports [41]. These disparities not only limit participation but also send a message about the perceived value of girls’ involvement in sports, reinforcing stereotypes and perpetuating inequities.

### 1.3. Research Problem

While considerable attention has been given to the health risks associated with climate change, the specific intersection between climate change and adolescent girls’ sport participation remains underexplored. Adolescent girls face distinct physiological vulnerabilities (e.g., lower sweating efficiency and heightened sensitivity to cold), sociocultural constraints (e.g., discomfort with uniforms and sweating), and institutional disadvantages (e.g., underfunded programs), all of which are likely to be amplified by climate-related disruptions [3,29,31].

To explore this issue, we conducted a scoping review to examine how climate-related stressors impact girls’ involvement in sports. The Ecologism and Individual × Environment (I×E) frameworks will subsequently be applied to interpret the findings, map interactions between environmental stressors and gender-specific barriers, and propose targeted intervention strategies.

## 2. Scoping Review

### 2.1. Methods

The scoping review follows the methodology proposed by Arksey and O’Malley [43] and the updated version by Levac et al. [44]. The protocol has been registered on Open Science Framework (Registration URL: https://osf.io/bcaj7, accessed on 30 September 2025).

#### 2.1.1. Search Strategy

This review draws on a detailed, comprehensive search of four databases (PubMed, Scopus, SPORTDiscus and Web of Science) that was conducted between the dates of 30 August to 15 September 2025, using the search terms listed in Table 1. A gray literature search on Google was also conducted. Search strings were modified for each database, using Boolean operators to combine terms.

#### 2.1.2. Citation Management

After completing both the peer-reviewed literature search and gray literature search, the results were exported into EndNote. In the table and abstract screening stage and full-text screening, each article was individually screened by J.H and S.D based on the inclusion and exclusion criteria (see Table 2). Conflicts were resolved through a discussion between the two reviewers.

#### 2.1.3. Inclusion Criteria and Screening

The authors applied pre-determined inclusion and exclusion criteria (see Table 2). Studies were not excluded based on geographical region due to the global impact of climate change. Studies that were not available in English were excluded.

#### 2.1.4. Data Extraction

Data extraction was captured in a Word document. Bibliographic information, objective of the study, methodological approach, geographic location, sample size and demographics were noted. Other data that was extracted included the physical activity and climate change variables and key findings.

### 2.2. Results

**Figure 1:**
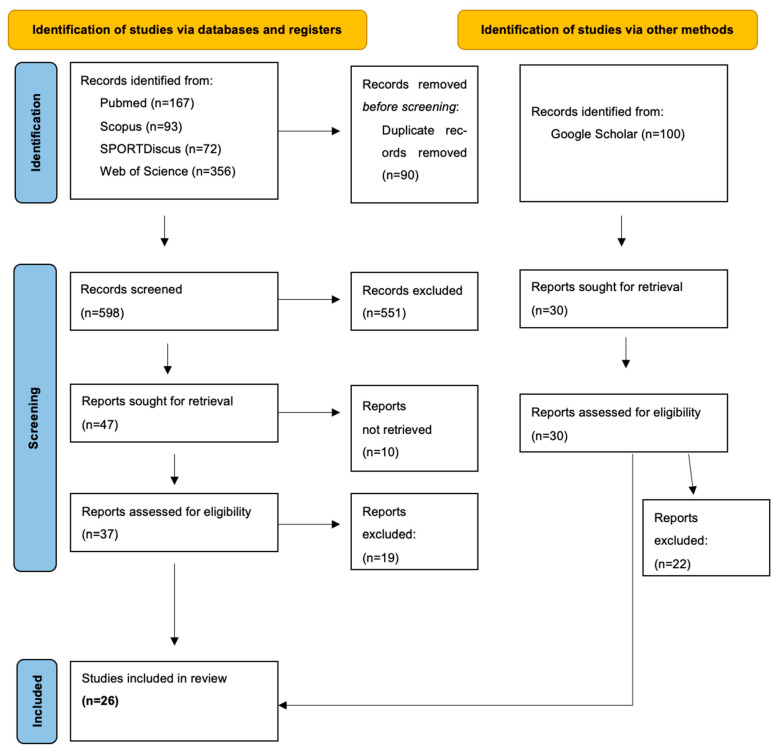
PRISMA Flow Diagram.

A total of 26 studies published between 1985 and 2025 were included (Figure 1, PRISMA flow diagram), the majority appearing after 2000 (*n* = 22, 85%), reflecting growing interest in climate and physical activity.

Study designs included laboratory experiments (*n* = 3), observational studies (*n* = 10), cross sectional analyses (*n* = 5), qualitative studies (*n* = 2), and reviews (*n* = 6). Research was geographically diverse, spanning Asia (*n* = 11), North America (*n* = 14), Europe (*n* = 10), Oceania (*n* = 10), and Central America (*n* = 1).

Study characteristics, including design, location, population, climate exposures, and physical activity outcomes, are summarized in Table 3. The findings are organized below according to the main climate factors examined: heat and temperature, rainfall and precipitation, air pollution, and daylight and seasonality.

#### 2.2.1. Climate Factors

##### Heat and Temperature

Heat and temperature were among the most frequently examined climate variables. Multiple studies demonstrated that higher ambient temperatures elevated physiological strain.

Aragon-Vargas et al. [45] observed body weight losses of up to 4.5% during a triathlon in extreme heat, with younger adolescent girls reporting more dizziness and discomfort than boys. Although dehydration rates were similar between sexes, only girls showed performance impairments, suggesting developmental or behavioral vulnerabilities. Similarly, Katsuur et al. [46] found that adolescent girls exhibited higher heart rates and lower stroke volumes than boys in hot conditions, indicating greater cardiovascular strain and perceived exertion that may contribute to earlier fatigue and reduced participation.

In contrast, other studies found no major sex-based differences. Hebestreit and Bar-Or [47] observed a linear rise in heart rate with increasing ambient temperature during both intermittent and continuous exercise, with boys and girls reacting similarly. McGarr et al. [48] likewise demonstrated that prolonged outdoor play elevated core body temperatures above 38 °C in both sexes, without sex-specific differences in heat strain. Consistent findings were reported by Morrison and Sims [49], who found no differences in sweat loss, core temperature, physiological strain index, or thermal sensation. Falk [50] found that prepubertal girls had lower sweat rates, reducing cooling efficiency, while puberty introduced hormonal effects that may blunt sweating in girls but enhance it in boys. Giersch and Charkoudian [51] added that girls and women generally exhibit lower blood pressure, greater vasodilation, and less sweat per gland compared with males, with these responses being context dependent, smaller body size may aid heat dissipation in dry conditions but increase vulnerability in humid or high-radiation environments. These findings are similar to Topham et al. [52]. Topham et al. [53] further noted no sex differences in core temperature or whole-body sweat loss in uncompensable heat, but did find regional sweat distribution differences, with girls secreting a greater proportion of sweat from the extremities.

Activity monitoring revealed that temperature effects often varied by sex: Duncan et al. [54] reported that a 10 °C increase in mean temperature moderately increased weekend step counts for boys but had only trivial effects for girls. Lewis et al. [55] found sedentary time was positively associated with maximum temperature in boys only, though Canadian data showed associations for both sexes. Patnode et al. [56] also found that higher temperatures modestly increased boys’ activity but not girls’. Qualitative evidence reinforces these patterns, with adolescents in Rajaraman et al. [57] identifying heat as a deterrent to participation. Broader epidemiological data link heat with elevated risk of exertional illness: Yard et al. [58] estimated over 6500 annual heat illness cases among U.S. high school athletes, most in football but also in girls’ sports such as soccer, softball, and volleyball. Similarly, Iwashita [59] found that school-based heatstroke incidents in Japan clustered during peak summer heat, with girls disproportionately affected in poorly ventilated gymnasiums during club activities.

##### Rainfall and Precipitation

Rainfall consistently emerged as a barrier to physical activity in children and adolescents. Quantitative studies demonstrated clear reductions in activity levels associated with precipitation. Duncan et al. [54] reported that moderate rainfall reduced daily step counts by up to 16%, with declines similar across day types and grade groups, but proportionally greater for girls. According to Kharlova et al. [60], every millimeter of rainfall corresponded to a 10 min reduction in physical activity, with girls more affected than boys due to their baseline deficit of five fewer active minutes per day. Belanger et al. [61] showed that every 10 mm of rainfall was associated with a 2–4% reduction in adolescent physical activity, while Chan and Ryan [62] found that rainfall reduced daily steps by 1700 for boys but had a greater impact on girls, whose activity dropped by 2300 steps with additional rainfall.

Findings across different contexts further confirm rainfall’s negative influence. Lewis et al. [55] reported that in Australia, physical activity was negatively associated with rainfall for both sexes, while maximum temperature effects were specific to girls. Kharlova et al. [60] also emphasized that girls’ overall physical activity was significantly lower than boys’ and that rainy conditions amplified this disparity, disproportionately reducing girls’ activity.

Qualitative evidence complements these quantitative findings. Rajaraman et al. [57] found that Canadian adolescents identified snow, rain, and extreme weather as barriers to participation. Similarly, Niven et al. [63] observed that adverse weather discouraged girls’ participation in physical education, underscoring how environmental barriers intersect with social and institutional ones.

##### Air Pollution

Air quality was another barrier to physical activity. Early evidence from Avol et al. [64] showed that ozone exposure caused modest but measurable declines in lung function during exercise, with girls reporting more non-respiratory symptoms. Gao et al. [65] demonstrated that prolonged exposure to air pollution reduced VO2max in children and adolescents, with girls experiencing greater declines in cardiorespiratory fitness than boys. While physical activity in less polluted districts improved girls’ VO2max, this benefit disappeared in high-pollution district areas, underscoring gender-specific vulnerability. More recent work has confirmed these disparities. Lee et al. [66] confirmed that air pollution was perceived as a major barrier in South Korea, with only 44% of adolescents rating outdoor air as acceptable; girls were less likely than boys to meet physical activity guidelines under these conditions.

##### Daylight and Seasonality

Several studies examined how day length and seasonal variation influence physical activity. Overall, longer daylight hours and warmer months were associated with higher levels of activity, while winter and shorter days reduced participation. Importantly, girls were consistently less active across all conditions and often showed sharper seasonal declines than boys.

Kharlova et al. [60] found that longer daylight reduced sedentary time and increased physical activity, yet girls remained less active than boys regardless of day length. Similarly, Gracia-Marco et al. [67] and Belanger et al. [61] reported that girls in colder climates engaged in less physical activity and more sedentary activity in winter compared with warmer months, while boys’ activity was less affected.

Seasonal differences were reinforced by Ridgers et al. [68], who observed that children overall spent less time in physical activity during summer than in winter. Girls engaged in more physical activity in autumn compared with winter, but no significant seasonal changes were observed for boys [68]. In the same study, Australian girls’ physical activity decreased in spring and summer relative to winter, while boys’ activity remained stable [68]. Silva et al. [69] further highlighted gendered patterns: boys’ physical activity rose sharply in summer but dropped in winter, whereas girls’ physical activity stayed relatively constant year-round. Tucker and Gilliland [70] reported that considerably more females were insufficiently active in winter compared with summer, while males maintained higher vigorous and adequate activity levels during colder months.

## 3. Theoretical Frameworks

### 3.1. Overview of Ecologism

Ecologism is a theoretical perspective that emphasizes the interconnectedness between humans and their surrounding environment, advocating for a holistic understanding of how ecological systems impact health, well-being, behavior, and social structures [71,72,73,74].

In the context of adolescent girls’ sports participation, ecologism draws attention to how rising temperatures, air pollution, and the loss of green spaces compromise the safety and availability of outdoor sports environments [24,75,76].

It also underscores the vulnerability of underfunded programs, which often lack shaded facilities, cooling systems, or resilient indoor venues—especially in low-income or underserved communities [20,31]. These disparities are amplified for girls’ teams, which are historically deprioritized in funding and climate adaptation planning. By framing sport settings as interconnected ecosystems, ecologism helps evaluate both environmental impacts and systemic inequities, offering a foundation for sustainable, inclusive infrastructure solutions.

### 3.2. Overview of the I×E Framework (Individual × Environment)

The Individual × Environment (I×E) framework explains how health outcomes emerge from the interplay between intrinsic vulnerabilities (I) (e.g., age, sex, physiology, health conditions) and extrinsic environmental stressors (E) (e.g., climate, pollution, socioeconomic conditions [17]. Rather than acting in isolation, these factors interact dynamically across the lifespan—for example, an adolescent’s asthma may be worsened by both genetic susceptibility (I) and air pollution (E) [77].

The I×E framework also highlights resilience, suggesting that protective factors (like stronger immune function or access to cooling infrastructure) can buffer risk, while compounding vulnerabilities increase it. For adolescent girls, intrinsic risks such as lower sweating efficiency and higher asthma prevalence intersect with extrinsic barriers like reduced access to climate-resilient sports facilities [25]. These interactions can heighten performance limitations and health risks, particularly in hot or polluted conditions. Applied to climate and sport, the I×E framework offers a structured lens to analyze how biological sensitivity and environmental constraints jointly influence girls’ participation. This can inform more precise, equity-focused intervention strategies [78,79].

### 3.3. Rationale for Framework Selection

The combination of ecologism and the I×E framework provides a comprehensive lens for examining the multifaceted impacts of climate change on adolescent girls’ participation in sports. Ecologism analyzes how systemic and environmental disruptions such as loss of green space or extreme weather events affect the infrastructure and accessibility of sports environments [72,73,80]. It emphasizes the importance of sustainable solutions, such as creating climate-resilient infrastructure and protecting green spaces, to address the broader environmental determinants of health [71,72,80].

**Figure 2:**
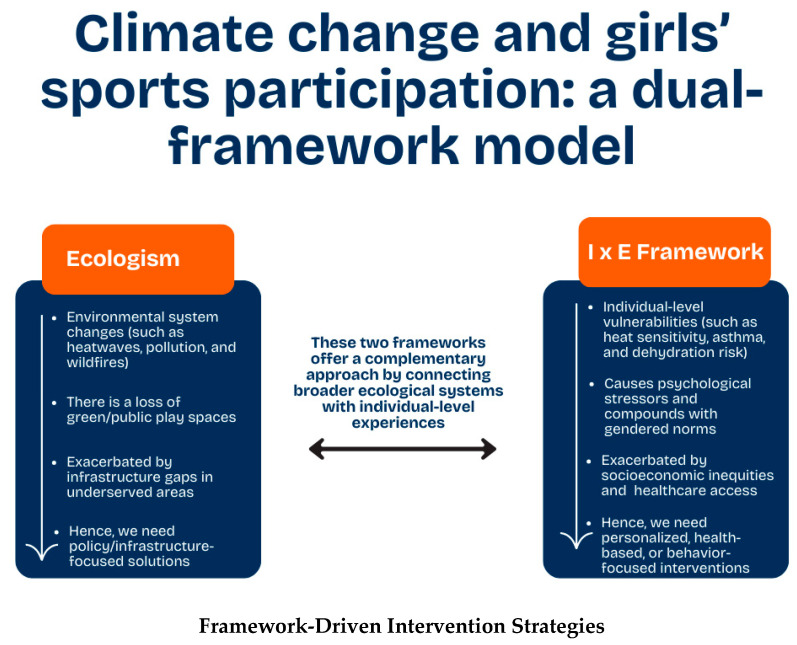
Climate change and girls’ sports participation: a dual-framework model.

Conversely, the I×E framework focuses on the interaction between individual characteristics and specific environmental conditions [17]. Together, these frameworks complement each other by bridging the gap between ecological systems and individual experiences (see Figure 2).

By integrating these perspectives, the interplay of climate change and individual-specific factors that influence adolescent girls’ participation in sports can be understood, thereby informing comprehensive interventions to address all dimensions of the issue. The resulting strategies can help mitigate the impact of climate change, promoting sustained participation in sports for girls (see Table 4).

### 3.4. Ecologism-Informed Interventions

#### 3.4.1. Adaptation of Sports Infrastructure

To address environmental challenges posed by climate change, ecologism advocates for sustainable and adaptive modifications to sports infrastructure. Urban greening initiatives, such as planting trees around sports fields, create natural shade to mitigate heat exposure while improving air quality through the filtration of pollutants [17,24]. These green spaces also promote biodiversity and ecological balance. Additionally, integrating sustainable cooling technologies in indoor sports facilities, such as solar-powered air conditioning or encouraging breaks in physical activity [21], can provide safe environments for physical activity during extreme weather events.

From the included studies within our scoping review, several ecologism-informed interventions were discussed. Indoor environments emerged as an area of concern as elevated rates of heat illness among female students in poorly ventilated gymnasiums pointed to the need for improved ventilation, active temperature monitoring, and adjustments to activity intensity [59]. Complementary qualitative work showed that overcrowding and lack of privacy in physical education spaces discouraged girls’ participation, suggesting that facility design improvements may enhance both safety and engagement [63].

##### Ecosystem-Based Approaches

Ecosystem-based approaches focus on leveraging natural systems to build resilience against climate extremes. For example, constructing natural barriers, such as wetlands or green belts, can provide flood protection and air purification for communities hosting outdoor sports activities. Designing multifunctional community spaces that incorporate climate-resilient features, such as permeable surfaces to reduce waterlogging and open areas that double as recreational and ecological zones, ensures usability even under adverse environmental conditions [17,24]. Based on our scoping review, we recommend prioritizing these nature-integrated solutions in communities where adolescent girls’ access to climate-resilient sports infrastructure is limited. Such interventions not only mitigate environmental stressors but also promote safer, more inclusive participation in sport.

#### 3.4.2. I×E Framework-Informed Interventions

##### Individual-Level Strategies

The I×E framework highlights the interaction of intrinsic factors and environmental factors, emphasizing the need for tailored interventions at the individual level. Educational programs targeting parents, coaches, and adolescent girls can play a pivotal role in navigating climate change resilience practices such as scheduled hydration, air quality awareness, and the use of UV-protective gear (see [81], for example). Social and cultural supports also play a critical role—studies from the scoping review found that parental encouragement, peer support, and access to equipment had stronger associations with participation than weather variables, particularly for girls [56]. Meanwhile, barriers like restrictive uniforms and safety concerns highlight the need for gender-sensitive approaches [57,63]. Structured hydration strategies were repeatedly emphasized, with evidence showing that girls are especially vulnerable to under-hydration during physical activity, reinforcing the importance of accessible water, scheduled drinking, and electrolyte options [45,49]. These individual-level efforts can protect girls from environmental stressors and empower them to continue participating in sports actively, despite climate-related challenges.

##### Environmental-Level Strategies

Environmental-level interventions include climate-conscious scheduling to reduce exposure to heat and weather extremes. Studies in our scoping review recommended rescheduling activities during high-risk conditions, avoiding peak heat hours, and increasing rest breaks to prevent heat-related illnesses [58,59]. Seasonal and weather-responsive strategies were frequently discussed. Observational studies documented lower physical activity levels in colder months or during rainfall, with sharper declines among girls [61,67,68]. Recommended strategies included expanding access to indoor alternatives, offering structured programming during inclement weather or reduced daylight, and maximizing outdoor opportunities under favorable conditions [55,60]. Additionally, investing in shaded outdoor facilities or climate-controlled indoor spaces can further ensure safe environments for sports participation, even during extreme weather conditions [29].

##### Integrated Interventions

Combining individual and environmental approaches can result in holistic interventions that address both intrinsic vulnerabilities and extrinsic barriers. For example, after-school programs held in climate-controlled facilities can provide safe, consistent spaces for sports while offering education on climate resilience [82]. These initiatives align with the I×E framework’s goal of fostering interactions between protective factors at both individual and environmental levels [17].

## 4. The Integration of Ecologism & I×E Framework-Informed Strategies

To effectively address the challenges of climate change and equitable sports participation, integrating ecologism-informed and I×E framework-based interventions offers a holistic approach. Because climate impacts vary by region—where heat and UV exposure may be more pressing in some areas, while others face greater challenges from precipitation or flooding—interventions must be tailored to specific environmental conditions [1,3,24]. Ecologism emphasizes sustainable adaptations to sports infrastructure, such as urban greening, natural cooling systems, and ecosystem-based designs, that not only mitigate environmental stressors but also enhance biodiversity and resilience. Other examples of what regional authorities can implement include shaded outdoor courts and promoting active transport to sports venues. Complementing this, the I×E framework underscores the importance of tailoring interventions to individual and environmental contexts. Educational programs can empower adolescent girls with climate resilience skills, while climate-conscious scheduling and the use of shaded or climate-controlled facilities ensure safe participation in sports. Adolescent girls should be encouraged and engaged in co-designing their sports environments, such as choosing eco-friendly gear and learning more about how climate affects their bodies and sports performance. Furthermore, there can be a focus on mentorship, where local female athletes are encouraged to mentor adolescent girls who share similar backgrounds and interests. These local female athletes can provide suggestions on how to personalize training to accommodate climate-related health needs, such as adapting training schedules to accommodate the climate and family and school commitments. Together, these strategies promote adaptive sports environments that support both ecological sustainability and individual well-being.

## 5. Discussion

There were several important gaps evident across the included studies. First, while most studies included mixed-sex cohorts, few provided detailed analyses of adolescent girls aged 10–19 years. This limits the ability to draw firm conclusions about gender-specific vulnerabilities. Second, the majority of studies were cross sectional or experimental in design, with few longitudinal investigations capable of capturing long-term patterns of participation or cumulative exposure to climate stressors. Seasonal studies [61,67,69] provided valuable snapshots but lacked follow-up across multiple years. Third, interventions were rarely evaluated. While many studies proposed strategies such as hydration guidance, improved ventilation, or weather-responsive programming, none systematically tested their effectiveness in real-world settings with adolescent girls. Also, the possibility of publication bias should also be considered as it is possible that research may have been less likely to report nonsignificant results, and such bias could influence the overall interpretation of trends observed in this review. Finally, certain climate stressors remain underexplored. While heat, precipitation, and seasonality were well represented, few studies examined the impacts of extreme events such as flooding or storms, or the compounded effects of multiple stressors (e.g., heat plus air pollution). Overall, the evidence base suggests that while climate change-related barriers to sport and physical activity are recognized, research remains limited in its focus on adolescent girls, its methodological breadth, and its testing of mitigation strategies.

Overall, our paper helps to address this gap by highlighting the need for implementing Ecologism and I×E framework-informed interventions to ensure all youth have the opportunity to engage in sports. Examples of interventions include climate-conscious scheduling of activities and the construction of natural barriers. These interventions should be implemented in partnership with local public health units and municipal planners, to ensure sustainability of the interventions and consideration of the local context. Moreover, these efforts contribute to broader public health and environmental sustainability goals, reinforcing the importance of implementing climate change-resilient strategies to protect population health and well-being.

Further research is essential to assess the long-term effectiveness of specific intervention strategies, such as climate-conscious educational programs, in promoting sustained sports participation, particularly among adolescent girls. Working in partnerships with stakeholders, such as school boards and local sports teams, can ensure longitudinal tracking is implemented in order to assess the long-term effectiveness of interventions. Furthermore, it is important to utilize qualitative methods (such as interviews and focus groups) to explore the perspectives of individuals directly involved in sports to understand their experiences with these interventions and gather their recommendations for improvement. Additionally, research across diverse geographical regions is needed, as the impacts of climate change vary significantly by location. By doing so, evidence-informed and context-sensitive solutions can be developed to promote health equity and resilience to climate change.

## 6. Conclusions

Climate change poses significant challenges to girls’ sports participation by creating and exacerbating environmental, individual, and social barriers. The rising prevalence of extreme weather events, poor air quality, and deteriorating sports infrastructure disproportionately affects adolescent athletes, particularly girls, who already face high dropout rates. These challenges highlight the critical need for framework-driven interventions, such as those informed by ecologism and the I×E framework. By leveraging these frameworks, a better understanding of the interplay between systemic ecological changes and individual vulnerabilities can be achieved, allowing for the design of interventions that not only mitigate these impacts but also promote equitable access to sports opportunities. While climate change impacts all youth athletes, adolescent girls experience unique and compounded risks due to physiological differences, sociocultural constraints, and institutional inequities. Addressing these gender-specific challenges through targeted adaptation strategies is essential to ensuring that climate-resilient sport environments are both inclusive and equitable.

## Figures and Tables

**Table 1:** Search Strategy.

Concept	Terms
Climate Change	“Climate change*” OR “Global warming” OR “Climate crisis” OR “Extreme heat” OR “Extreme cold” OR “Heatwave*” OR “Heat wave*” OR “Heat stress*” OR “High temperature*” OR “Rising temperature*” OR “Thermal stress” OR “Air pollution” OR “Poor air quality” OR “Wildfire*” OR “Wildfire smoke” OR “Particulate matter” OR “Ozone exposure” OR “Extreme weather” OR “Flood*” OR “Drought*”OR “Natural disaster*” OR “Climate hazard*” OR “Environmental hazard*” OR “Weather variability” OR “Environmental exposure*” OR “Environmental stressor*” OR “Ecological risk*”
Adolescent girls	“girl*” OR “female*” OR “pediatric*” OR “child” OR “adolescen*” OR “prepubescen*” OR “pre-pubescen*” OR “pubescen*” OR “school aged” OR “elementary school*” OR “middle childhood” OR “middle school*” OR “teenage*” OR “teen*” OR “youth*” OR “young women*” OR “young woman*” OR “young adult” OR “school age” OR “schoolchild*” OR “juvenile*”
Sports participation	“sport*” OR “athlet*” OR “exercise*” OR “acrobat*” OR “alpine*” OR “archer*” OR “badminton*” OR “baseball*” OR “basketball*” OR “baseball*” OR “biath*”OR “bmx*” OR “bobsle” OR “boccia*” OR “boxing*” OR “boxer*” OR “bowling” OR “bowler*” OR “broomball*” OR “canoe*” OR “cheerlead*” OR “cricket*” OR “crossfit*” OR “cross-fit* OR “curling*” OR “cross-country” OR “cycling” OR “cyclist” OR “diving*” OR “diver*” OR “equest*” OR “fencing*” OR “fencer*” OR “field hockey” OR “football*” OR “futsal*” OR “golf*” OR “goalball*” OR “gymnast*” OR “handball*” OR “hockey*” OR “judo” OR “karate*” OR “kayak*” OR “kickbox*” OR “kick-box*” OR “lacross*” OR “lawn bowl*” OR “luge*” OR “martial* art*” OR “muay thai” OR “mountain* bik*” OR “netball*” OR “pentath*” OR “racquet*” OR “ringette*” OR “rock climb*” OR “rockclimb*” OR “rower*” OR “rowing*” OR “rugby*” OR “runner*” OR “running*” OR “sailing*” OR “sailor*” OR “soccer*” OR “ski jump*” OR “ski mountain*” OR “skiing*” OR “skier*” OR “skateboard*” OR “skating*” OR “skater*” OR “sledding*” OR “snowboard*” OR “softball*” OR “speedskat*” OR “speed-skat*” OR “speed* skat*” OR “squash*” OR “swim*” OR “surfing*” OR “surfer*” OR “taekwondo*” OR “tennis*” OR “track field” OR “trampolin*” OR “triath*” OR “volleyball*” OR “wakeboard*” OR “water polo*” OR “wrestling*” OR “wrestler*” OR “weightlight*” OR “weight lift*” OR “jiu jitsu*” OR “jiu-jitsu*” OR “jogging*” OR “jogger*” OR “kendo*” OR “kung fu*” OR “kung-fu*” OR “mountaineer*” OR “qigong*” OR “tai ji” OR “tai chi*” OR “taiji*” OR “taichi*” * *Was used in our search strategy to find variations of a word by searching for its root or stem*

**Table 2:** Inclusion and Exclusion Criteria.

Domain	Inclusion	Exclusion
Publication Information	Published in English Research published in a peer-reviewed journal or a gray literature source Primary research or review	Conference abstracts and proceedings, theses, commentaries, news articles etc. Conceptual/theoretical papers
Target Population	Adolescent girls (10–19 years old)	Primary focus beyond adolescent girls Papers that included all genders but did not perform a subgroup analysis on adolescent girls were excluded
Outcomes of Interest	Focus on either weather (such as temperature or precipitation) or climate change Included physical activity as a variable (i.e., sports participation or levels of physical activity)	Primary focus beyond weather and climate change Does not focus on physical activity levels

**Table 3:** Characteristics of included studies (*n* = 26).

Author, Year	Location	Population	Study Design	Climate/Environmental Exposure	Physical Activity Outcome
Aragon-Vargas, 2013 [45]	Costa Rica	Adolescent Triathletes ages 9–17	Observational	Extreme heat	Dehydration, Performance
Katsuura, 1985 [46]	Japan	19 children (10 boys, 9 girls) age 10–11	Experimental	Extreme heat	Physiological strain (e.g., heart rate, stroke volume)
Hebestreit, 1998 [47]	Germany and Canada	20 children (12 boys, 8 girls), age 8–11 years	Experimental	Extreme heat	Physiological strain (e.g., heart rate, VO_2_max)
McGarr, 2020 [48]	Canada	18 children (7 girls, 11 boys) age 10–14	Observational	Extreme heat	Physiological strain
Morrison, 2014 [49]	Global	Pre-pubertal children age 4–12; includes boys and girls	Review	Extreme heat	Physiological strain, Dehydration
Falk, 1998 [50]	Global	Children and adolescents (various ages, prepubertal to post pubertal), compared with adults.	Review	Extreme Heat Extreme Cold	Thermal responses, Physiological strain (e.g., sweating, body fat)
Giersch, 2025 [51]	Global	Women (primarily premenopausal, healthy, active individuals)	Review	Extreme heat	Physiological strain (e.g., blood pressure, vasodilation, sweating)
Topham, 2022 [52]	Global	Children and adults Girls age 8–13 Boys age 10–14	Review	Extreme heat	Physiological strain (e.g., sweating)
Topham, 2024 [53]	Australia	17 boys, 18 girls age 10–16	Observational	Extreme heat	Physiological strain (e.g., sweating)
Duncan, 2008 [54]	New Zealand	1115 children (536 boys, 579 girls), age 5–12 years	Observational	Extreme heat, Rainfall	Physical activity participation (step counts)
Lewis, 2016 [55]	Canada and Australia	491 Australia children 524 Canadian children Age 9–11	Observational	Extreme heat, Rainfall	Physical activity participation, Sedentary time
Patnode, 2010 [56]	USA	294 adolescents age 10–17	Observational	Precipitation (snow, ice, hail)	Physical activity participation
Rajaraman, 2015 [57]	India and Canada	India: age 14–15 Canada: age 11–18	Qualitative	Weather, Extreme heat, Extreme cold, Precipitation	Physical activity participation and experience
Yard, 2010 [58]	USA		Cross sectional	Extreme heat	Heat illness, Dehydration
Iwashita, 2018 [59]	Japan	Secondary and high school students (2005–2014). 3819 heatstroke cases reported; 2204 occurred on school grounds.	Observational	Extreme heat	Heat illness (heatstroke)
Kharlova, 2020 [60]	Norway	2015 children (1020 girls, 995 boys), age 6–12	Cross sectional	Daylight, Extreme heat, Precipitation	Moderate to Vigorous Physical Activity, Sedentary time
Belanger, 2009 [61]	Canada	1293 students age 12–13	Cross sectional	Seasons, Rainfall	Physical activity participation
Chan, 2009 [62]	Global	Adolescents	Review	Rainfall	Physical activity participation
Niven, 2014 [63]	UK	38 girls, age 13–16	Qualitative	Weather	Physical education experience
Avol, 1987 [64]	USA	66 children age 8–11	Experimental	Ozone exposure (air pollution)	Lung function, respiratory and non-respiratory symptoms
Gao, 2013 [65]	China	2048 school children (age 8–10 years old; 51.6% boys, 48.4% girls).	Cross sectional	Air pollution	Cardiorespiratory fitness
Lee, 2023 [66]	South Korea	Adolescents age 12–18	Cross sectional	Air Pollution	Environmental perceptions, Sedentary behavior, Active transport
Gracia-Marco, 2013 [67]	Europe	2173 adolescents age 12.5 to 17.5	Observational	Seasons	Physical activity participation, Sedentary time
Ridgers, 2015 [68]	Australia	326 children age 8–11	Observational	Seasons	Physical activity participation
Silva, 2011 [69]	Portugal	24 students (12 boys, 12 girls), age 11.04 ± 1.45	Observational	Seasons	Physical activity participation
Tucker, 2007 [70]	Global	Adolescents age 12–16	Review	Seasons	Physical activity participation

**Table 4:** Climate-related barriers to girls’ sports participation and framework-informed recommended interventions.

Barrier Type	Examples	Recommendations
Environmental	Heatwaves Poor air quality Loss of green space Storm/flood damage	Urban greening Climate-resilient infrastructure Shaded/indoor facilities Sustainable cooling technologies
Physiological	Heat sensitivity Asthma Eczema Dehydration UV exposure	Hydration training UV-protective gear Climate conscious scheduling Climate education Access to medical screening
Social	Underfunded programs Gender bias Unsafe timing Reduced prioritization	Equity-focused funding Evening scheduling adjustments Inclusive policy reforms Participatory infrastructure design

## Data Availability

No new data were created or analyzed in this study. Data sharing is not applicable to this article.

## Abbreviation

The following abbreviation is used in this manuscript:

I×E	Individual × Environment
